# Effect of n‐3 polyunsaturated fatty acid on bone health: A systematic review and meta‐analysis of randomized controlled trials

**DOI:** 10.1002/fsn3.2655

**Published:** 2021-11-29

**Authors:** Yuqi Dou, Ying Wang, Zekun Chen, Xue Yu, Defu Ma

**Affiliations:** ^1^ School of Public Health Peking University Health Science Center Haidian District, Beijing China

**Keywords:** bone formation, bone mineral density, bone resorption, meta‐analysis, polyunsaturated fatty acids

## Abstract

Bone metabolism is a complicated process, which involves bone modeling and remodeling. If this process is unbalanced, bone loss and resultant osteoporosis might occur. Recently, nutrition supplementations such as n‐3 polyunsaturated fatty acids (PUFAs) are considered to be used on improving the bone metabolism and reducing the risk of osteoporosis. To more precisely assess the effects of n‐3 PUFA supplementation on bone mass and clarify its potential mechanism, we have conducted a systematic review and meta‐analysis. Based on the strict inclusion and exclusion criteria, 12 articles were included in this meta‐analysis. The results in articles show that n‐3 PUFAs could slightly enhance the level of bone mineral density (BMD) (0.005 g/cm^2^; 95% CI, 0.000–0.010) (*n* = 7), which was the primary outcome for the research in comparison with the control group. In addition, the results also illustrate that the increasing effect on BMD (0.024 g/cm^2^; 95% CI, 0.020–0.028) became more significant for postmenopausal women. N‐3 PUFAs had no significance on the level of bone‐specific alkaline phosphatase (BALP) (−0.24 µg/L; 95% CI, −0.86 to 0.39) and osteocalcin (−0.63 μg/L; 95% CI, −1.84 to 0.57) (*n* = 5), which are the specific markers of bone formation. When compared with the eicosapentaenoic acid + docosahexaenoic acid supplementation, the supplementation form of α‐linolenic acid significantly increased the content of BALP (0.396 µg/L; 95% CI, 0.069–0.724). The effects of n‐3 PUFAs on bone resorption biomarkers containing type I collagen cross‐linked C‐terminal peptide (CTX) and type I collagen cross‐linked N‐terminal peptide (NTX) are considered and used in our study. Results indicated that participants who received n‐3 PUFAs significantly decreased the level of CTX in the human body (−0.367 μg/L; 95% CI, −0.726 to −0.007) (*n* = 4). However, there was no significant difference in NTX levels in humans after supplementation with n‐3 PUFA (−1.744 µg/L; 95% CI, −3.970–0.481) (*n* = 3). For postmenopausal women, it presented a significant decreasing level of CTX (−0.393 µg/L; 95% CI, −0.651 to −0.135) and NTX (−2.082 µg/L; 95% CI, −2.970 to −1.195) within their bodies. In conclusion, these findings suggested that n‐3 PUFAs might have a beneficial effect on bone health, especially for α‐linolenic acid supplementation form or for postmenopausal women.

## Graphical abstract

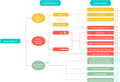

## 1 INTRODUCTION

Osteoporosis is considered as one of the most prevalent skeletal disorders in the elderly population all over the world, especially in the group of postmenopausal women who are suffering from menopause due to estrogen deficiency (Riggs et al., 1998). Osteoporosis is characterized by low bone density, low bone strength, and micro‐architectural degradation of bone tissue, leading to enhanced bone fragility and a consequent increase in fracture and physical disability risk. It is estimated that there are approximately 9 million cases of fracture caused by osteoporosis in 2000 in the world, which leads to a huge economic burden. Only the incidence of hip osteoporotic fractures is expected to reach 6.3 million by 2050 (Cooper et al., 2011). Therefore, manners of preventing osteoporosis and reducing its prevalence are of great importance.

Bone metabolism is a complicated process involving bone modeling and remodeling. Bone remodeling occurs continuously and is mediated through the coupled cycle of bone formation and bone resorption. Due to the imbalance in this process, bone loss and resultant osteoporosis occur. Therefore, searching for a nutritional intervention or supplementation that could exert a profound influence on bone metabolism is imperative.

Recently, nutrition supplementations such as n‐3 polyunsaturated fatty acids (PUFAs), calcium, and vitamin D, and exercise have been put forward to improve bone metabolism and reduce the risk of osteoporosis (Tartibian et al., 2011; Vanlint & Ried, 2012). n‐3 PUFAs mainly include eicosapentaenoic acid (EPA), docosahexaenoic acid (DHA), and alpha‐linolenic acid (ALA). In the human diet, EPA and DHA are mostly obtained from fish and lesser from certain plants (Vanlint & Ried, 2012). Walnuts and flaxseed oil were the predominant sources of ALA (Cornish & Chilibeck, 2009). Several studies have demonstrated that n‐3 PUFAs play pivotal roles in anti‐inflammation, cardiovascular disease prevention, and improvement in mild cognitive impairment (Dangour et al., 2012; Jain et al., 2015; Molfino et al., 2014). It is widely admitted that n‐3 PUFAs would contribute to the growth and development of the central nervous system and visual function in infants (Guesnet & Alessandri, 2011). Moreover, the relationship between n‐3 PUFA supplementation and bone metabolism has been extensively examined recently (Fonolla‐Joya et al., 2016).

However, there is inconsistent evidence of randomized controlled trials (RCTs) concerning the efficacy of n‐3 PUFA supplementation on human bone health. Bassey et al. (2000) indicated that there were no changes in bone turnover markers, for both bone formation and resorption, between the supplementation of n‐3 PUFAs and control groups. However, Nawata et al. (2013) found that the intake of n‐3 fatty acids is positively correlated with bone mineral density (BMD) of the lumbar spine. Moon et al. (2012) demonstrated that high levels of EPA and DHA supplementation might reduce the risk of osteoporosis in Korean postmenopausal women. Salari et al. (2008) evaluated the effects of n‐3 PUFA supplementation on bone health and the prevention of osteoporosis by conducting a systematic review and showed that n‐3 PUFAs are beneficial for bone health and the prevention of osteoporosis.

With the aim to more precisely assess the effects of n‐3 PUFA supplementation on bone mass and to clarify its potential mechanism, we carried out this research by evaluating the change in bone turnover makers including bone formation, bone resorption, and BMD, which could reflect the metabolic activity of bone.

## 2 MATERIALS AND METHODS

### 2.1 Registration

A systematic review and meta‐analysis of randomized controlled trials was conducted according to the guidelines of Meta‐analysis of Randomized Controlled Trials in Epidemiology and Preferred Reporting Items for Systematic Reviews and Meta‐analyses (Hutton et al., 2015) and has been registered in the International Prospective Register of Systematic Reviews (PROSPERO) (CRD42018092107).

### 2.2 Search strategy

A detailed, pre‐specified protocol was used. A systematic documentation search without language limitation was performed till October 2020 using the electronic databases of PubMed, Embase, Cochrane Library with foreign articles, and China National Knowledge Infrastructure (CNKI) Chinese citation database, and Wanfang database with Chinese articles. The key terms used to search for articles were as follows: (“osteoporosis” OR “rarefaction” OR “bone rarefaction” OR “bone mineral density” (BMD) OR “bone mass” OR “bone mineral content” OR “osteocalcin” OR “bone‐specific alkaline phosphatase” (BALP) OR “BAP” OR “BSAP” OR “type I collagen cross‐linked N‐terminal peptide” (NTX) OR “type I collagen cross‐linked C‐terminal peptide”(CTX) and (“n‐3 fatty acid” OR “eicosapentaenoic acid” (EHA) OR “docosahexaenoic acid” (DHA) OR “fatty acid” OR “polyunsaturated fatty acid*” (PUFA*)). We also supplemented the process described above with a manual search of reference lists of related articles in this field to ensure a complete collection. If necessary, the authors of included studies were contacted if further information was required.

### 2.3 Inclusion and exclusion criteria

The inclusion criteria were followed: (1) The old who is over 50 years old took fatty acid for at least 1 month; (2) all types of RCTs; and (3) BALP, osteocalcin, NTX, CTX, and BMD were used as an index of bone metabolism. If the study sample was proven to overlap with another article, or if two articles described aspects of the same study, only the publication with the larger sample was selected. If the study contained several comparisons, we choose the maximum dose or the maximum follow‐up time of each research in the pooled meta‐analysis. Two authors independently searched all titles and abstracts for selection.

### 2.4 Data extraction and assessment of risk of bias

Two reviewers independently extracted the data of interest using a standardized spreadsheet. Discrepancies were resolved by consensus. The following data were extracted from each study: (1) name of the first author and year of publication; (2) location of the study; (3) population characteristics including sample size, age, and sex; (4) research information including supplementation method, duration, form, and dose; and (5) the final concentrations of various bone metabolic indices. Bone formation indicators (BALP and osteocalcin) and bone resorption indicators (CTX and NTX) were selected as secondary outcomes of this study. At the same time, BMD was chosen as the primary outcome of this research. The quality of the included RCTs was assessed according to the Cochrane Collaboration’s Tool (Higgins et al., 2011), including the method of random sequence generation, allocation concealment, blinding of participants and personnel, blinding of outcome assessment, incomplete outcome data, and selective reporting.

### 2.5 Statistical analyses

The meta‐analysis was performed using STATA metan (version 13.0; StataCorp). Statistical significance was considered at *p* value <.05, unless otherwise specified. Statistical heterogeneity among the studies was tested through Cochran's Q test. Inconsistency was tested using the *I^2^* test (very low: <25%, low: 25% to <50%, moderate: 50% to <75%, large: >75%). A *p* value <.10 was considered to be statistically significant. When the test for heterogeneity was statistically significant, the estimation of the weighted mean difference was calculated using the DerSimonian‐Laird (D‐L) random effect model. Means and standard deviations of the post‐intervention for the supplementation and control groups were combined as weighted mean difference with 95% confidence interval (CI).

The included studies contain not only EPA + DHA supplementation but also ALA supplementation. To ensure the consistency in comparison, the dose of ALA supplementation was converted to the dose of EPA + DHA according to Baker et al. (2016). In terms of the Academy of Nutrition and Dietetics, which recommends consuming approximately 0.5 g/day of EPA + DHA (Baker et al., 2016), we decided to choose 0.5 g/day as the possible threshold effect dose concentration to divide the study into low‐dose group (<0.5 g/day) and high‐dose group (≥0.5 g/day).

A subgroup analysis was carried out based on the following aspects: characteristics of the supplementation objects, supplementation duration, supplementation dose, supplementation type, region of conducting research and control group, and whether women using “estrogen replacement therapy” or “estrogen” are categorically excluded. A sensitivity analysis that was estimated by omitting one study in each turn was performed to investigate the influence of a single trial on the overall effect. A meta‐regression analysis was used to estimate the linear dose–response relationship among supplementation dose, supplementation duration, and intervention effect. At the same time, publication bias was also evaluated with funnel Egger’s linear regression test and Begg’s rank correlation test. In addition, we explored the publication bias by visual inspection of funnel plots.

## 3 RESULTS

### 3.1 Search results

**FIGURE 1:**
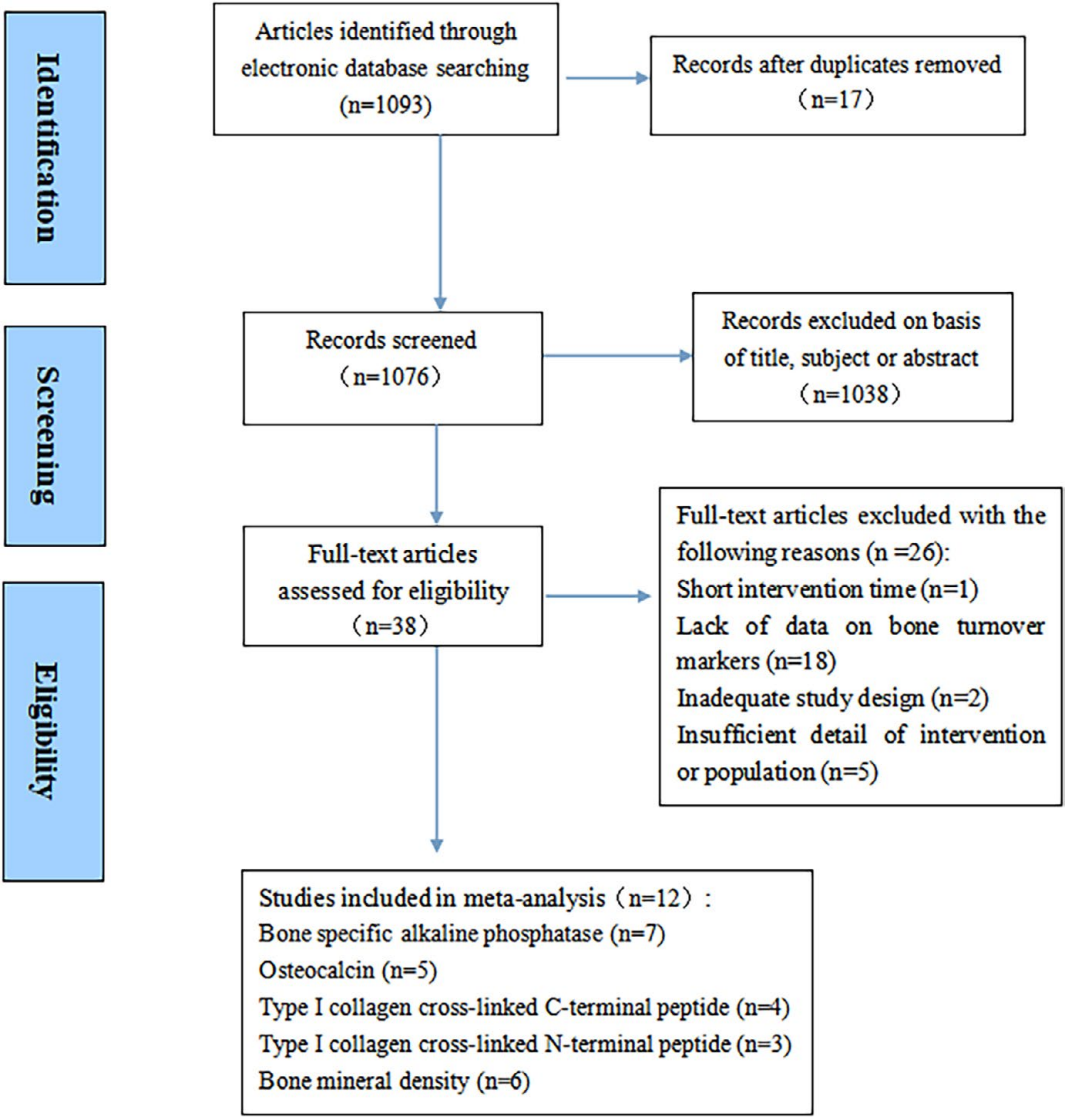
Flow diagram for the selection and exclusion of studies

To begin with, a total of 1093 relevant abstracts of articles were initially identified through a preliminary electronic and manual literature search, of which 17 full texts were excluded because of content repetition. Then, on caring about the eligibility and quality of the remaining articles, 1038 articles were removed after screening the titles, subjects, or abstracts. Besides, the rest 38 full‐text articles were inspected for a detailed estimation. Among these, one study was excluded because of having a very short follow‐up time (<1 month) and 18 articles were eliminated because of lacking statistical data about the related bone metabolic markers. What is more, two articles whose research design did not meet the requirements and five with insufficient data on intervention or population information were excluded. Finally, based on the strict inclusion and exclusion criteria, 12 articles were remained in this meta‐analysis. The selection process of the research is shown in Figure 1.

### 3.2 Characteristics of the included studies

The demographic characteristics of the included literature are listed in Table 1. The 12 articles that satisfied the eligibility criteria for this meta‐analysis were published from 1998 to 2016. Among the 12 articles, four of them were undertaken in America (Dong et al.,^2014^; Griel et al., 2007; Hutchins‐Wiese et al., 2014; Lappe et al., 2013) and two studies were conducted in South Africa (Bassey et al., 2000; Trebble et al., 2005) and Canada (Cornish & Chilibeck, 2009; Dodin et al., 2005), respectively. Four studies were performed in Australia (Vanlint & Ried, 2012), England (Kruger et al.,^1998^), Spain (Fonolla‐Joya et al., 2016), and Iran (Tartibian et al., 2011), respectively. The study participants were 50–79.5 years of age. In these studies, four comparisons had been intervened for <6 months and the intervention duration of the rest comparisons ranged from 6 to 36 months. The intervention dose of the included studies ranged from 0.3 to 14 g/day. Meanwhile, there was a large gap between different studies, which might be one source of clinical heterogeneity. Of these 12 articles, three studies used ALA as the form of intervention and the others used EPA + DHA as the main form of intervention. Only three articles did not specify in the exclusion criteria to exclude postmenopausal women using estrogen or estrogen replacement therapy, and these three articles were Dodin (Dodin et al., 2005) (BMD), Cornish (Cornish & Chilibeck, 2009) (BMD), and Fonolla (Fonolla‐Joya et al., 2016) (BALP).

**TABLE 1:** Characteristics of studies included in the meta‐analysis

Study (year)	Country	Age/Sex	Length of treatment (months)	Number (Int/Con)	Intervention	Indices
Bassey (2000)	England	56/F	12	19/24 21/21	Int: Ca + marine fish oil 0.44 g/day Con: Ca	BMD; osteocalcin; NTX; BALP
Cornish (2009)	Canada	65/F,M	3	14/14 11/12	Int: ALA 14 g/day Con: placebo	BMD
Dodin (2004)	Canada	54/F	12	85/94	Int: ALA 9 g/day Con: Placebo	BMD
Dong (2014)	America	75/F	6	77/39	Int:0.72 g EPA + 0.48 g DHA/day Con: placebo	BALP; Osteocalcin; NTX
Fonolla (2016)	Spain	60/F	12	63/54	Int: EPA + DHA 40 mg/100 ml Con: placebo	BALP
Griel (2007)	America	50/M	1.5	23/23	Int1: ALA 3.6 g/day Con: placebo Int2: ALA 6.5 g/day Con: placebo	NTX; BALP; CTX
Hutchins (2014)	America	62/F	3	20/18	Int: 2.52 g EPA + 1.68 g DHA/day Con: placebo	BALP; CTX
Kruger (1998)	South Africa	80/F	36	29/31 21/31	Int: EPA + DHA 0.4 g/day Con: placebo	BMD; BALP; osteocalcin
Lappe (2013)	America	55/F	6	30/28 31/33	Int: PUFAs 1 g Con: placebo	BALP
Tartibian (2011)	Iran	61/F	6	20/18	Int: 0.18 g EPA + 0.12g DHA/day Con: no treatment	BMD; osteocalcin; CTX
Van (1995)	South Africa	80/F	4	10/10	Int: 0.72 g EPA + 0.48 g DHA/day Con: 4 g olive oil	Osteocalcin
Vanlint (2011)	South Australia	59/F	12	19/18	Int: DHA 0.4 g/day Con: placebo	BMD; CTX

Abbreviations: ALA, α‐Linolenic acid; BALP, bone‐specific alkaline phosphatase; BMD, bone mineral density; Con, control group; CTX, type I collagen cross‐linked C‐terminal peptide; DHA, docosahexenoic acid; EPA, eicosapentaenoic acid; Int, intervention group; NTX, urinary type I collagen cross‐linked N‐terminal peptide; PUFA, polyunsaturated fatty acid.

### 3.3 Assessment of risk of bias

A risk of bias was evaluated to assess the methodological quality of all RCTs included in the meta‐analysis using the Cochrane Collaboration’s Risk of Bias tool, where “−,” “?,” and “+” are used to indicate high, unclear, and low risk of bias, respectively. All studies had a low risk on sequence generation. Nine of the 12 studies that did not report the method of allocation concealment had an unclear risk of selection bias. As for performance bias, eight of the 12 trials had a low risk of bias as the methodology for participant and personnel blinding was adequate. In addition, 10 of the 12 studies had an unclear risk of detection bias for not providing enough information to judge whether the blinding of outcome assessment was undertaken. At the same time, 11 of the 12 studies illustrate full data and hold a low risk of attrition bias. Ten of the 12 trials were considered to be at low risk of reporting bias, as all pre‐specified outcomes from their protocols were included in the articles. The overall assessment on each risk of bias is presented in Figure S1. The quality of evidence for all outcomes was moderate.

### 3.4 Bone formation markers

**FIGURE 2:**
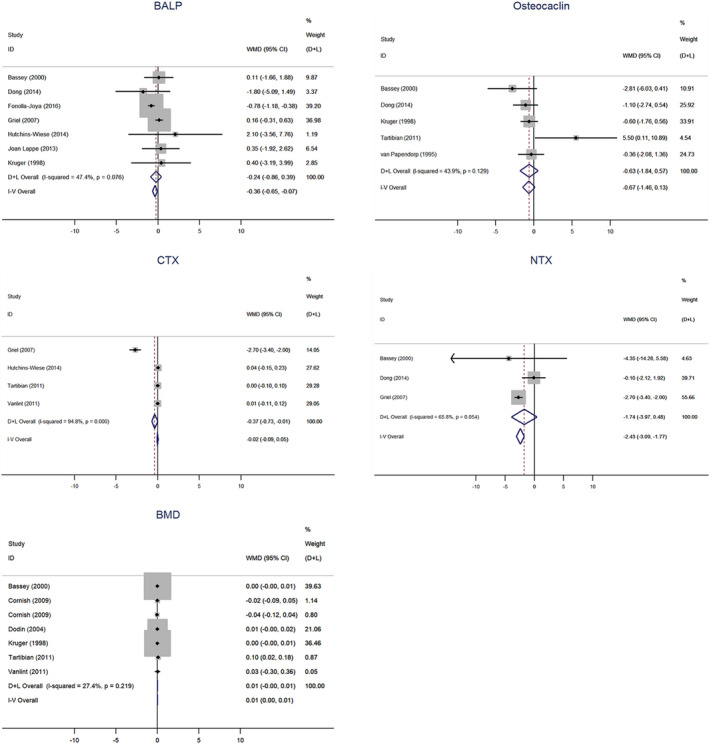
Forest plot for assessing changes in bone‐specific alkaline phosphatase (BALP), osteocaclin (OC), type I collagen cross‐linked C‐terminal peptide (CTX), urinary type I collagen cross‐linked N‐terminal peptide (NTX) and bone mineral density (BMD) with polyunsaturated fatty acid supplements. The diamond denotes overall treatment effect with 95 % confidence interval (CI) and weighted mean difference (WMD)

The specific markers of bone formation measured in our meta‐analysis are BALP and osteocalcin (Figure 2; Table 2). In our meta‐analysis, seven studies analyzed the influence of n‐3 PUFA supplementation on BALP. Meanwhile, n‐3 PUFAs did not show a significant effect on the level of BALP (−0.237 µg/L; 95% CI, −0.863 to 0.389).

**TABLE 2:** Subgroup analysis for bone turnover markers

Subgroup analysis	WMD for BALP (95%CI)	WMD for OC (95%CI)	WMD for CTX (95%CI)	WMD for NTX (95%CI)	WMD for BMD (95%CI)
Supplement duration					
<6 months	0.409 (0.086, 0.732)	1.503 (−3.428, 6.434)	−1.033 (−1.700, −0.367)	−2.400 (−2.988, −1.182)	−0.028 (−0.061, 0.02)
≥6 months	−0.709 (−1.085, −0.334)	−0.659 (−2.355, 1.037)	0.003 (−0.073, 0.078)	−0.268 (−2.244, 1.708)	0.024 (0.020, 0.028)
Dose class					
<0.5g/day	0.023 (−0.707, 0.753)	−0.855 (−1.951, 0.240)	−2.400 (−2.988, −1.812)	−2.405 (−2.899, −1.911)	0.024 (0.020, 0.028)
≥0.5 g/day	0.242 (−1.064, 1.547)	0.789 (−1.571, 3.148)	0.003 (−0.044, 0.051)	−0.100 (−2.116, 1.916)	0.049 (−0.006, 0.104)
Supplement type					
EPA + DHA	−0.648 (−1.016, −0.280)	−0.333 (−1.705, 1.039)	0.003 (−0.044, 0.051)	−0.268 (−2.244, 1.708)	0.026 (0.022, 0.030)
ALA	0.396 (0.069, 0.724)	/	−2.400 (−2.988, −1.812)	−2.400 (−2.988, −1.812)	0.008 (−0.005, 0.021)
Region					
Developed country	0.009 (−0.753, 0.772)	−1.450 (−2.910, 0.009)	0.001 (−0.048, 0.050)	−2.082 (−2.970, −1.195)	0.006 (0.000, 0.012)
Developing country	0.260 (−0.967, 1.487)	0.704 (−1.335, 2.743)	−1.565 (−3.504, 0.374)	/	0.037 (0.032, 0.043)
Control					
Placebo control	0.387 (0.071, 0.704)	0.489 (−1.624, 2.601)	−0.393 (−0.651, −0.135)	−2.031 (−2.995, −1.067)	0.033 (0.020, 0.028)
Other control	−0.738 (−1.123, 0.352)	−0.901 (−2.417, 0.614)	/	−4.350 (−14.277, 5.577)	0.005 (−0.002, 0.012)
Estrogen or estrogen replacement therapy					
Unused	0.145 (−0.288, 0.578)	−0.333 (−1.705, 1.039)	−0.393 (−0.651, −0.135)	−2.082 (−2.970, −1.195)	0.349 (0.051, 0.648)
Not clear	0.017 (−0.194, 0.228)	/	/	/	0.040 (−0.533, 0.612)

Abbreviations: ALA, α‐Linolenic acid; BALP, Bone‐specific alkaline phosphatase; BMD, bone mineral density; CI, confidence interval, CTX, type I collagen cross‐linked C‐terminal peptide; DHA, docosahexenoic acid; EPA, eicosapentaenoic acid; NTX, urinary type I collagen cross‐linked N‐terminal peptide; OC, osteocalcin; WMD, weighted mean difference.

The subgroup analysis showed that the increasing effect of n‐3 PUFA supplementation on the content of BALP became more significant (0.409 µg/L; 95% CI, 0.086–0.732) when the supplement duration was <6 months. Besides, the participants who received ALA supplementation indicated a significant increase in BALP (0.396 µg/L; 95% CI, 0.069–0.724) within their bodies. The subgroup analysis with respect to control type suggested that in comparison with other control, the effect was significant in a placebo‐controlled trial (0.387 µg/L; 95% CI, 0.071–0.704).

Five studies reported values of osteocalcin before and after n‐3 PUFA intervention. However, no significant differences were observed in osteocalcin for overall effect and subgroup analysis. In addition, sensitivity analyses with each study excluded individually suggested that no individual study had a significant influence on the pooled results of BALP and osteocalcin, and it is shown in Figure S2.

### 3.5 Bone resorption markers

The effects of n‐3 PUFAs on bone resorption biomarkers including CTX and NTX were taken into account in our study (Figure 2; Table 2). The CTX changeable data are available in four studies. The pooled weighted difference in means (−0.367 µg/L; 95% CI, −0.726 to −0.007) indicated that participants who received n‐3 PUFAs significantly decreased the level of CTX.

The subgroup analysis by duration revealed that short‐term interventions whose duration was <6 months experienced a significant reduction in CTX (−1.033 µg/L; 95% CI, −1.700 to −0.367). Likewise, the decreasing effect on CTX became significant (−2.400 µg/L; 95% CI, −2.988 to −1.812) after excluding studies whose supplementation doses exceeded 0.5 g/day. Results of the subgroup analysis with supplementation type indicated the decreasing effect in CTX became significant with ALA supplementation in comparison with the EPA + DHA supplementation (−2.400 µg/L; 95% CI, −2.988 to −1.812). Besides that, the subgroup analysis also found that placebo‐controlled study had a lower level of CTX (−0.393 µg/L; 95% CI, −0.651 to −0.135).

There are (Bassey et al., 2000; Dong et al.,^2014^; Griel et al., 2007) that reported the results about the level of NTX in the meta‐analysis. No significant difference in NTX was observed after n‐3 PUFA supplementation (−1.744 µg/L; 95% CI, −3.970–0.481). However, in the subgroup analysis, n‐3 PUFA supplementation also showed a significant declining effect on NTX after rejecting studies whose supplementation duration was more than 6 months (−2.400 µg/L; 95% CI, −2.988 to −1.182). Compared with participants in the high‐dose group, participants whose supplementation dose was <0.5 g/day underwent a significant reduction in NTX (−2.405 µg/L; 95% CI, −2.899 to −1.911). Results of sub‐analyses also indicated that studies with ALA supplementation showed a significant decrease in NTX relative to EPA + DHA group (−2.400 µg/L; 95% CI, −2.988 to −1.812). Likewise, the decreasing effect on NTX became significant (−2.031 µg/L; 95% CI, −2.995 to −1.067) after removing the studies that were not placebo‐controlled study.

Furthermore, sensitivity analyses after excluding the male study by Griel et al. indicated that postmenopausal women presented a significant decreasing level of CTX (−0.393 µg/L; 95% CI, −0.651 to −0.135) and NTX (−2.082 µg/L; 95% CI, −2.970 to −1.195) (Figure S2).

### 3.6 BMD

Seven studies reported BMD index (Figure 2; Table 2), which was the primary outcome for the research, and there was a significant increase in BMD with n‐3 PUFA supplementation (0.005 g/cm^2^; 95% CI, 0.000–0.010). The subgroup analysis with respect to supplementation dose demonstrated that in comparison with high‐dose supplementation, participants whose supplementation dose was <0.5 g/day had minor increase in BMD (0.024 g/cm^2^; 95% CI, 0.020–0.028). Also, participants in the EPA + DHA group presented with a mild increase in BMD relative to those in the ALA supplementation (0.026 g/cm^2^; 95% CI, 0.022–0.030). Meanwhile, an unobvious improvement in BMD was observed in placebo‐controlled study (0.033 g/cm^2^; 95% CI, 0.020–0.028). Furthermore, sensitivity analyses showed that after excluding studies whose participants were not postmenopausal women, the increasing effect on BMD in postmenopausal women became significant (0.024 g/cm^2^; 95% CI, 0.020–0.028) (Figure S2). Besides, n‐3 PUFAs significantly increased BMD levels in postmenopausal women who did not use estrogen or estrogen replacement therapy (0.349 g/cm^2^; 95% CI, 0.051–0.648); However, no significant increase was observed in BMD levels in postmenopausal women who may have used estrogen or estrogen replacement therapy (0.040 g/cm^2^; 95% CI, −0.533–0.612).

### 3.7 Meta‐regression

To clarify whether there was a linear dose–response or linear time–response relationship among the supplementation dose, the intervention duration, and the effects on bone markers, meta‐regression analysis was performed. However, no statistical significance was found in our analysis (Figures S3 and S4).

### 3.8 Publication bias

We performed Egger’s and Begg’s tests to evaluate a potential publication bias. Both tests did not find any significant publication bias (BALP: Egger's and Begg's tests were *p* = .571 and *p* = .652, respectively. BMD: Egger’s and Begg’s tests were *p* = .961 and *p* = .881, respectively. Osteocalcin: Egger’s and Begg’s tests were *p* = .560 and *p* = .624, respectively. NTX: Egger’s and Begg’s tests were *p *= .722 and *p* = .602, respectively. CTX: Egger’s and Begg’s tests were *p *= .135 and *p* = 1, respectively). Meanwhile, the funnel plot showed that the distribution was basically symmetrical (Figure S5).

## 4 DISCUSSION

To the best of our knowledge, this is the first study to clarify the effect of n‐3 PUFA supplementation on bone turnover markers for the elder, including bone formation indices, bone resorption indices, and bone mass indices, by performing a meta‐analysis. The current study found that n‐3 PUFA supplementation could significantly decrease the level of bone resorption index (CTX) and increase the level of BMD in comparison with the control group. Besides, if the supplementation form was ALA, the interventional duration was <6 months or the study population was postmenopausal women, the improvement effect of n‐3 PUFA supplementation for osteoporosis was more significant.

Shen et al. (2017) conducted a meta‐analysis to investigate the effect of omega‐3 fatty acids on bone turnover markers in postmenopausal women. However, this study did not include all relevant published articles according to its inclusion criteria. One study focusing on the effect of dietary n‐3 fatty acids on bone resorption markers was not included in this meta‐analysis. Besides, to more accurately evaluate the change in bone metabolism, we not only selected indicators of bone formation and bone resorption but also analyzed the BMD. Abdelhamid et al. (2019), which included in the articles before 2017, also conducted a systematic review and meta‐analysis to assess the effects of increasing dietary omega‐3 on the risk of fractures. The article by Salari et al. (2010), which was included in the article, did not specify the specific type of omega‐3 or details of the placebo group, so this article was excluded from our study. Besides, due to the different inclusion criteria between that article whose participants were greater than or equal to 40 years of age and our study whose participants were greater than or equal to 50 years of age, the study by Chen et al. (2016) was excluded. In addition to that, the inclusion criteria for the article specified that only studies with an intervention duration >6 months were included, which inevitably reduced the number of relevant studies. Because of the small number of articles and the limited data, the six omega‐3 trials have been determined to have a low overall bias risk, and it is not possible to determine the effect of omega‐3 on bone mass. Furthermore, the authors also did not check all of them and missed five articles (Bassey et al., 2000; Dong et al., 2014; Fonolla‐Joya et al., 2016; Kruger et al., 1998; Lappe et al., 2013), whose intervention duration was >6 months.

Bone turnover marker concentrations in blood and urine could reflect bone metabolism activity (Glendenning et al., 2018). Markers of bone turnover are usually divided into bone formation and bone resorption markers. Bone formation markers are generated by osteoblasts or derived from pro‐collagen metabolism, whereas bone resorption markers are the degradation products of osteoclasts or collagen (Christenson, 1997). The imbalance between bone formation and bone resorption leads to net bone loss. BALP and osteocalcin, considered as the appropriate and specific indicator for bone formation, are widely used in clinical research (Lumachi et al., 2009; Tamaki et al., 2013). Meanwhile, the measurement of bone resorption markers, especially for CTX and NTX produced by type I collagen, has been proven to be valuable for the evaluation of osteoporosis (Szulc, 2012; Vasikaran et al., 2011). The present meta‐analysis showed that n‐3 PUFA supplementation might have a profitable influence on inhibiting bone resorption, which led to a reduction in CTX level. Besides, BMD was chosen as the primary index in our study reflecting bone mass, which measures the accumulation of metabolic activity over time. The result of this meta‐analysis suggested that n‐3 PUFA supplementation significantly increased the level of BMD in relation to the control group. Farina et al. (2011a) demonstrated that high intakes (≥3 servings/week) of fish oil relative to lower intakes were positively associated with maintenance of femoral neck BMD.

Interestingly, this meta‐analysis suggested that supplementation type had a vital effect on bone formation and bone resorption. Our the subgroup analysis found that ALA could significantly increase the level of BALP and lower the level of CTX and NTX, but EPA + DHA supplementation had no significant influence on BALP, CTX, and NTX. Farina et al. (2011b) indicated that no significant associations were observed between intakes of EPA + DHA and hip fracture risk in the combined sample of men and women, but higher ALA intake was associated with lower hip fracture risk. Participants in the highest quartile of ALA intake had a 54% lower risk of hip fracture than those in the lowest quartile. Rajaram et al. (2017) also showed that there was a significant negative association between serum CTX and red blood cell membrane ALA (*p* = .047), but the negative association was not observed between serum CTX and red blood cell membrane DHA, which speculated that the form of supplementation could exert significant influence on bone resorption. In a word, ALA might have a greater effect on bone metabolism in comparison with EPA + DHA. Further research is needed to explore the association between different forms of supplementation and bone turnover markers and verify whether ALA can be supplemented to prevent bone loss.

After rejecting studies whose supplementation duration was more than 6 months, n‐3 PUFA supplementation showed a significant declining effect on CTX (−1.033 µg/L; 95% CI, −1.700 to −0.367) and NTX (−2.400 µg/L; 95% CI, −2.988 to −1.182) as well as an increasing effect on BALP (0.409 µg/L; 95% CI, 0.086 to 0.732) in human bodies. However, it was worth noting that a small significant increasing effect on BMD was observed after rejecting studies whose supplementation duration was <6 months. Bone turnover markers change earlier than BMD, and thus, bone turnover markers may be employed to monitor response to therapy before changes in BMD become apparent (Shen et al., 2017). It may take a long time to detect the changes in BMD, which makes it a challenge to encourage patients’ adherence. Our results suggested that n‐3 PUFA supplementation may have short‐term effects for bone turnover markers and long‐term effects for BMD.

In addition, placebo‐controlled studies showed that PUFAs supplementation significantly reduced the levels of NTX and CTX and increased the level of BALP and BMD in comparison with other types of control, which indicated that other substances may affect the function of PUFAs supplementation on bone health. Previous randomized controlled trials also suggested that there were no significant differences between the fish oil intervention group and the control group, which was composed of calcium in total body BMD or markers of bone turnover (Bassey et al., 2000). Accordingly, subsequent research should further analyze whether other related substances will affect PUFAs on bone resorption or bone formation.

This study had some limitations. First, significant heterogeneities were present in our analysis, which might be due to the different sample sizes, supplementation doses, gender of subjects, and age of participants. Second, to include more research data, the subgroup analysis used multiple n‐3 supplementation doses or different follow‐up time compared with the control group, and we could not avoid double counting (or triple counting) from these studies, which was the limitation of our research.

## 5 CONCLUSIONS

In summary, our results supported the presumption that n‐3 PUFAs may play a beneficial role on bone health by inhibiting bone resorption, as well as promoting bone formation and enhancing BMD, especially for ALA intervention form or for postmenopausal women. In addition, n‐3 PUFAs supplementations might have short‐term effects for bone turnover markers and long‐term effects for BMD. High‐quality RCTs with large sample sizes are needed to clarify the best supplementation dose, duration, and form for the effect of n‐3 PUFAs supplementation on bone metabolism in the future.

## CONFLICT OF INTEREST

The authors declare that there is no conflict of interest with respect to this research study and paper.

## Supporting information

Supplementary Material

## Data Availability

The datasets used or analyzed during the current study are available from the corresponding author on reasonable request.
