# Supplementary material for: Effect of n‐3 polyunsaturated fatty acid on bone health: A systematic review and meta‐analysis of randomized controlled trials
Source: Food Sci Nutr. 2021 Nov 29;10(1):145–54. doi: 10.1002/fsn3.2655 (PMC8751426; doi:10.1002/fsn3.2655)
Supplement: Supplementary file 1 — Supplementary Material [file FSN3-10-145-s001.docx]

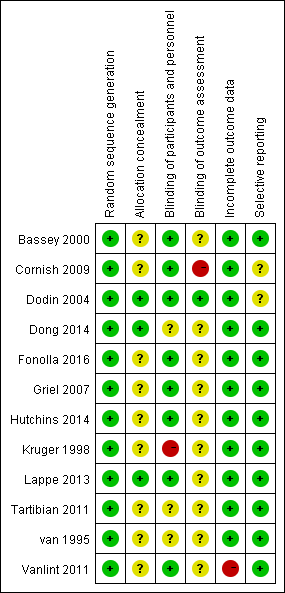

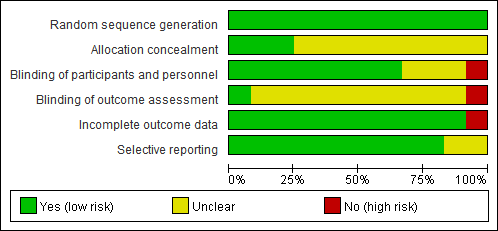


**Figure 1. Risk of bias assessment for literatures included in this meta-analysis**

**
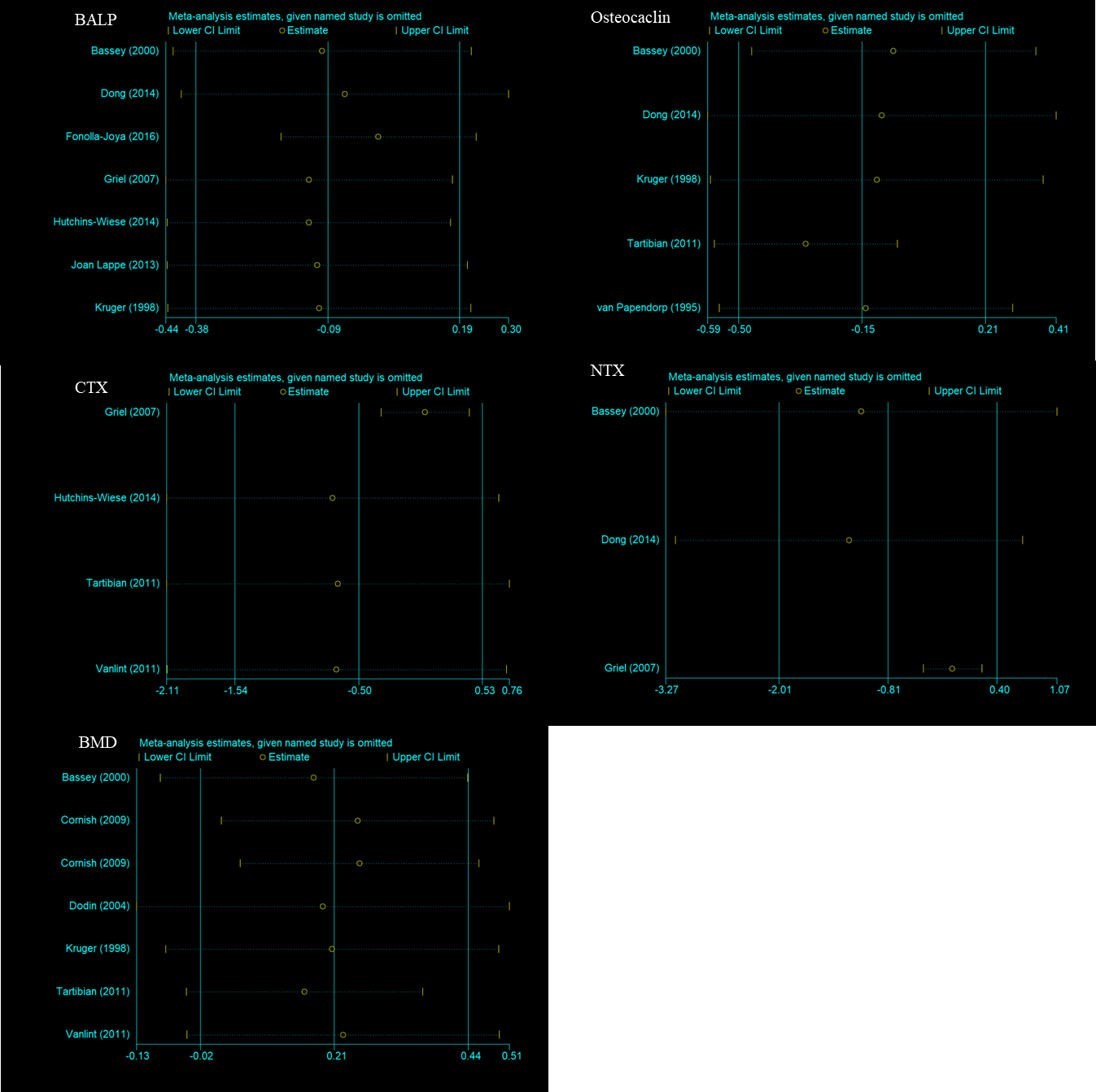
**

**Figure 2. Sensitivity analysis results for bone-specific alkaline phosphatase (BALP), osteocaclin (OC), type I collagen cross-linked C-terminal peptide (CTX), urinary type I collagen cross-linked N-terminal peptide (NTX) and bone mineral density (BMD).**

**
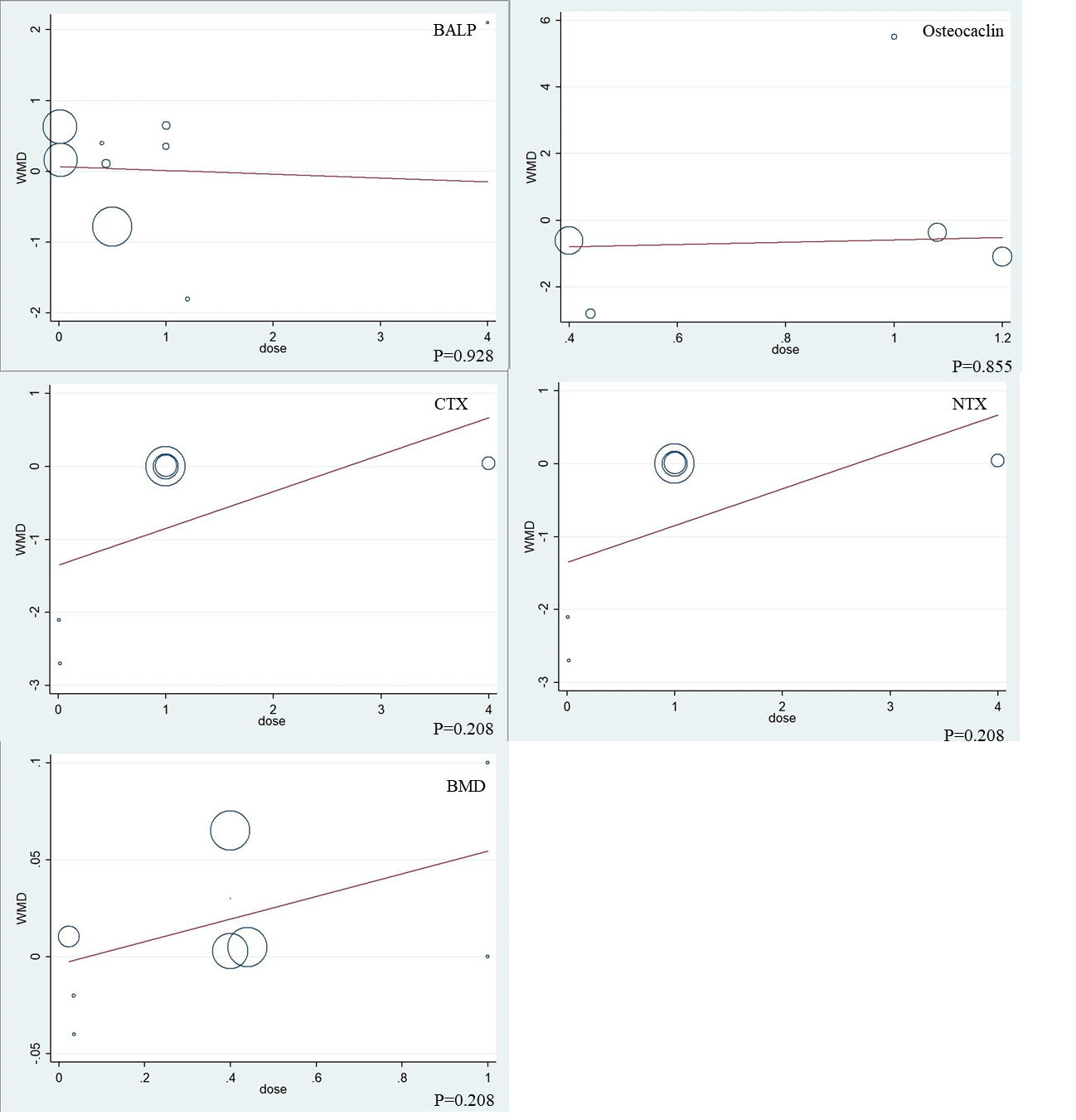
**

**Figure 3. Meta-regression analysis of the relationship between the supplement dose and the changes in bone-specific alkaline phosphatase (BALP),** **osteocaclin (OC), type I collagen cross-linked C-terminal peptide (CTX), urinary type I collagen cross-linked N-terminal peptide (NTX) and bone mineral density (BMD). Weighted mean differences of changes for bone turnover markers (y-axis) and supplement dose (x-axis) are shown. Weights of included trials were based on the inverse of the total variance and are shown by the size of the circles.**

**
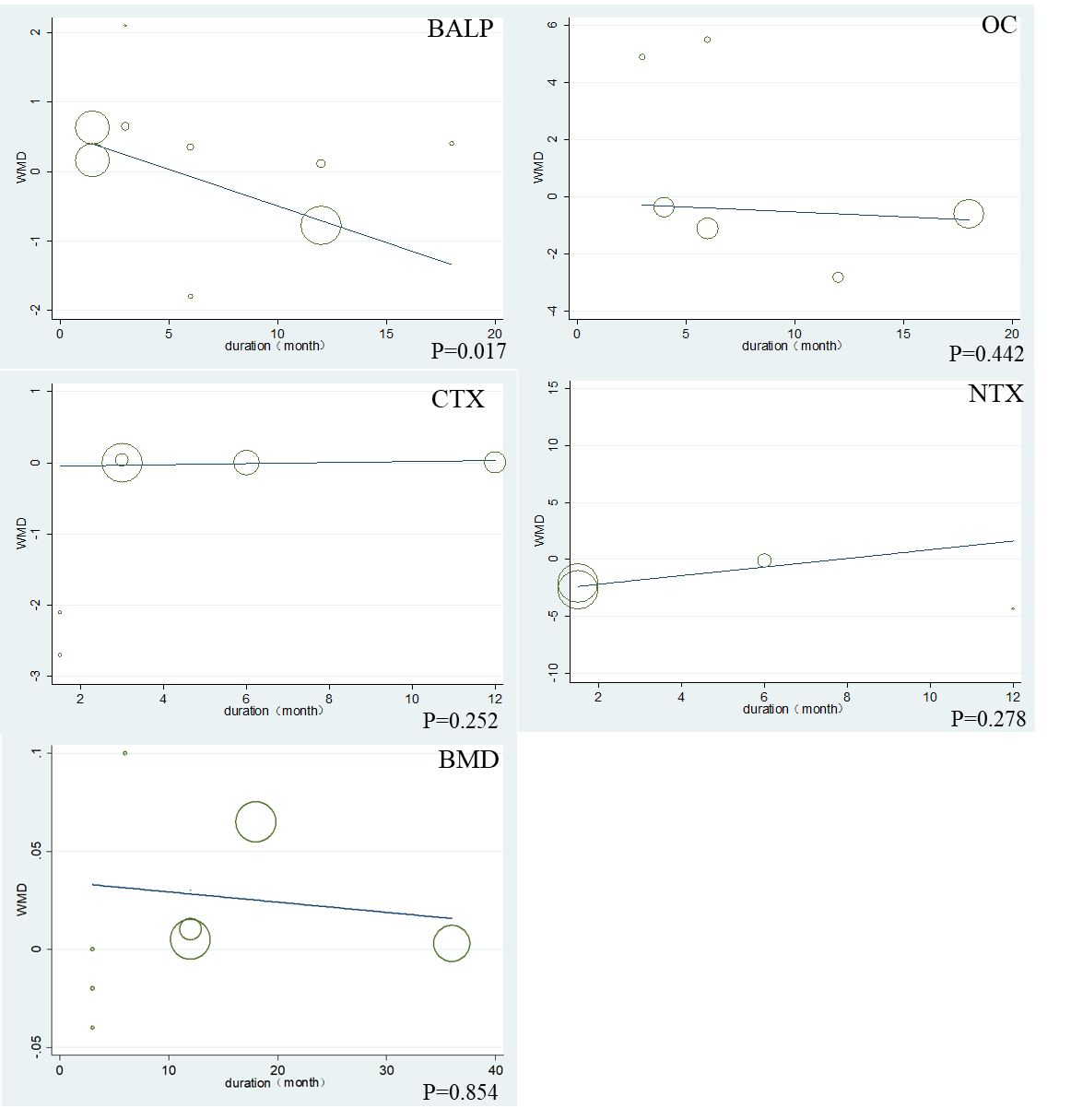
**

**Figure 4. Meta-regression analysis of the relationship between the intervention duration and the changes in bone-specific alkaline phosphatase (BALP), osteocaclin (OC), type I collagen cross-linked C-terminal peptide (CTX), urinary type I collagen cross-linked N-terminal peptide (NTX) and bone mineral density (BMD). Weighted mean differences of changes for bone turnover markers (y-axis) and intervention duration (x-axis) are shown. Weights of included trials were based on the inverse of the total variance and are shown by the size of the circles.**

**
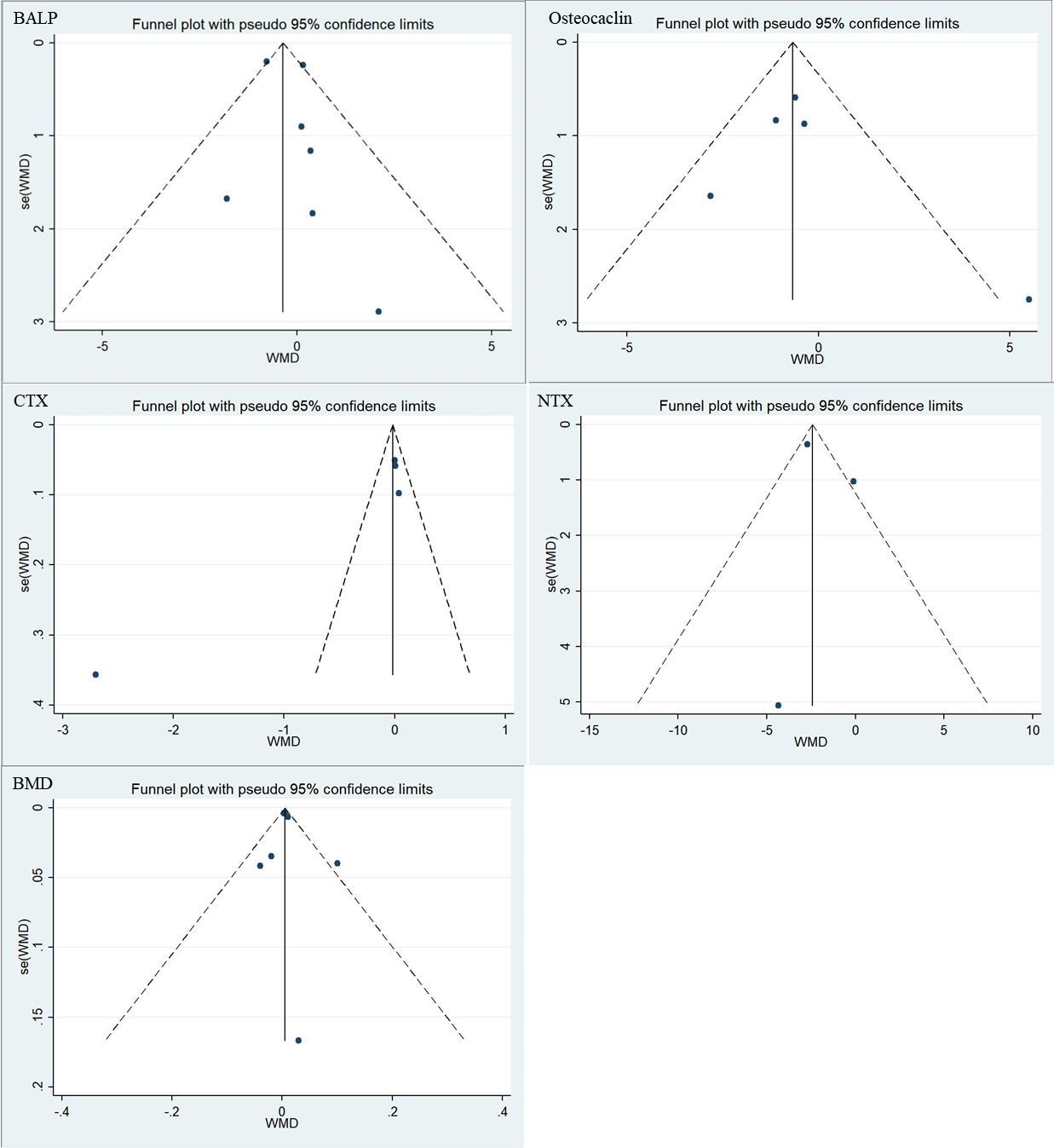
**

**Figure 5. Funnel plot for** **bone-specific alkaline phosphatase (BALP), osteocaclin (OC), type I collagen cross-linked C-terminal peptide (CTX), urinary type I collagen cross-linked N-terminal peptide (NTX) and bone mineral density (BMD).**
